# Alterations of prolyl endopeptidase activity in the plasma of children with autistic spectrum disorders

**DOI:** 10.1186/1471-244X-5-27

**Published:** 2005-06-02

**Authors:** Naghi Momeni, Berit M Nordström, Vibeke Horstmann, Hassan Avarseji, Bengt V Sivberg

**Affiliations:** 1 Department of Health Sciences, Autism Research, Lund University, Lund, Sweden; 2 Department of Neurology, Golestan University of Medical Science, Gorgan, Iran

## Abstract

**Background:**

Prolyl Endopeptidase (PEP, EC 3.4.21.26), a cytosolic endopeptidase, hydrolyses peptide bonds on the carboxyl side of proline residue in proteins with a relatively small molecular weight. It has been shown that altered PEP activity is associated with various psychological diseases such as schizophrenia, mania and depression. Autistic Spectrum Disorders (ASD) are neuropsychiatric and behavioural syndromes affecting social behaviours and communication development. They are classified as developmental disorders. The aim of this study was to examine the hypothesis that PEP activity is also associated with ASDs.

**Methods:**

Fluorometric assay was used to measure PEP activity in EDTA plasma in children with ASD (n = 18) aged 4–12 years (mean ± SD: 7.9 ± 2.5). These results were then compared to PEP activity in a control group of non-ASD children (n = 15) aged 2–10 years (mean ± SD: 6.4 ± 2.2).

**Results:**

An alteration in PEP activity was found in the children with ASD compared to the control group. There was much greater variation of PEP activity in the group of ASD children when compared to the controls (SD= 39.9 and SD 9.6, respectively). This variation was significant (p < 0.0005), although the mean level of PEP activity in the group of ASD children was slightly higher than in the control group (124.4 and 134.1, respectively).

**Conclusion:**

Our preliminary finding suggests a role for PEP enzyme in the pathophysiology of autism but further research should be conducted to establish its role in the aetiology of psychiatric and neurological disorders, including autism and related spectrum disorders.

## Background

Prolyl Endopeptidase (PEP, EC 3.4.21.26) is a cytosolic endopeptidase. PEP cleaves peptide bonds on the carboxyl side of proline residues in low molecular weight proteins containing the recognition sequence X-Pro-Y, where X is a peptide or protected amino acid and Y is an amide, a peptide, an amino acid, an aromatic amine or an alcohol [1]. PEP can only hydrolyse small peptides and is thought to be involved in the metabolism of hormones/neuropeptides. However, PEP also degrades many active hormones/neuropeptides, e.g. oxytocin, arginine vasopressin (AVP), substance P, neurotensin, luteinizing hormone-releasing hormone (LH-RH) and thyrotropin-releasing hormone (TRH) [2]. These low molecular weight proteins, particularly oxytocin, AVP, TRH, neurotensin, and substance P, profoundly affect social behaviour, emotions, stress level, responsivity, reward-seeking and positive reinforcement behaviour [3]. Altered PEP activity has been observed in psychiatric disorders such as depression, mania and schizophrenia [4].

Autistic Spectrum Disorders (ASD) are neuropsychiatric and behavioural syndromes affecting social and communicative development. They were classified as developmental disorders in DSM-IV (American Psychiatric Association 1994) [5]. Severe communication deficits and social and behavioural abnormalities often appear during the first three years of life but the diagnosis is often made later, due to a lack of resources. The aetiology of ASD is not yet known. Symptoms of ASDs are related to the abnormal functioning of certain centres within the brain: in particular the cerebellum, brain stem and limbic region [6]. ASDs are also associated with several specific dysfunctions including fragile X syndrome [7], a cascade of complex gene-environment interactions [8,9], hyperserotoninemia, [10,11], increased levels of opioid [12,13] and high levels of arginine-vasopressin (AVP) [our unpublished observation]. Low plasma levels of the neuropeptide hormone oxytocin have also been found in a group of children with ASD when compared to the normal age-matched controls [14].

Altered levels of the neuropeptide hormones oxytocin, arginine vasopressin and other related hormones/peptides may be a result of proteolytic enzyme activity such as PEP, which is involved in the formation and degradation of various neuropeptides. Our hypothesis is that altered activity of proteolytic enzymes, such as PEP, in children with ASD (children <12 years) might lead to the degradation of some specific neuropeptide hormones, affecting social behaviour and communication.

## Methods

### Materials

N-Benzyloxycarbonyl-glycyl-prolyl-4-methylcoumarinyl-7-amide (Z-Gly-Pro-4-methylcoumarinyl-7-amide) was obtained from Bachem in Bubendorf, Switzerland. Dithiothereitol, ethylenediamineteraacetic acid disodium salt dihydrate (EDTA), benzamidinium chloride, p-chloromercuribenzoate (PCMB) and pepstatin, sodium azide, dithiothereitol (DTT) were obtained from Sigma in St. Louis, Missouri USA. Acetic acid and 1,4-dioxan were obtained from Merk in Darmstadt, Germany. A Perkin Elmer Fluorimeter LS 50B was used to determine the release of 7-amino-4-methylcoumarine at excitation and emission wavelengths of 370 nM and 440 nM.

### Subjects

Eighteen ASD and a control group of 15 non-ASD children participated in this study. The children with ASD were selected from children attending rehabilitation centres in Sweden. The original diagnosis of ASD was made jointly by a psychiatrist and psychologist who made the diagnosis in accordance with DSM IV (APA, 2000) and the International Classification of Diseases (ICD) (WHO, 1993). Their diagnosis was then independently confirmed by the specialist in autism spectrum disorders at Lund university hospital. As a routine measure, these children undergo health checks including a dental examination. This requires the children to be anaesthetised, since many children with ASD are unable to understand what is required of them and incapable of cooperating when a dental examination is carried out. During our research period 18 ASD children underwent this examination. Wechsler Intelligence Scale for Children (WISC) was used to estimate the children's functional abilities. The ASD group consisted of 14 boys and four girls ranging from 4 to 12 years (mean 7.9 years; SD, 2.5). Information about additional dysfunction and medication was not available. Paediatricians at the children's hospital selected the children in the control group when they came to the hospital to be treated for various physical conditions. None of the children in the control group had any mental disabilities. The control group (non-ASD) consisted of nine boys and seven girls ranging from 2–10 years (mean 6.4 years; SD, 2.2), (Table 1).

**Table 1:** PEP activity (fluorescence intensity unit) of EDTA plasma and age in control (n = 15) and ASD groups (n= 18).

**Study groups**		**Percentiles %**	**Fluorescence intensity**	**Child's age**
			**intesity**	
***Control group***				
	Mean		124.4	6.4
	Std. Deviation		9.6	2.2
	Minimum		105.4	2
	Maximum		144.0	10
	Percentiles	25	115.8	5
		50	125.6	7
		75	130.7	8
				
***ASD group***				
	Mean		134.1	7.9
	Std. Deviation		39.9	2.5
	Minimum		48.1	4
	Maximum		201	12
	Percentiles	25	109.3	6
		50	147.6	8
		75	160.2	10

The Ethics Committee at the Faculty of Medicine, Lund University, approved this study (LU-70-00).

### Sample collection

Venous blood from ASD children was collected under general anaesthesia when they were undergoing another medical treatment. This was done in the presence of a child psychiatrist with special training in the field of childhood psychosis. Venous blood from a control group of non-ASD children was collected in evacuated 4 mL EDTA tubes, containing 0.084 ml of 0.34 M K_3_-EDTA solution. These tubes (Vacutainer System) were obtained from Becton-Dickinson Inc., Plymouth, UK. Plasma from EDTA-containing blood was produced immediately after collection by centrifugation at 1300 g for 10 min at 4°C. 30 μL of cocktail inhibitors per 1 mL plasma was then added to the produced plasma sample. The inhibitor cocktail stock solution used was Tris 2.0 M, Na-EDTA 0.9 M, Benzamidin 0.2 M, E-64,10 μM and Pepstatin 48 μM. The PEP activity of the samples was analysed immediately after the production of plasma. The remaining samples were stored for further investigation at -70°C.

### Assay procedure

The method used to assay the PEP using the hydrolysis of the fluorogenic substrate (Z-Gly-Pro-4-methylcoumarinyl-7-amide) has previously been described by Momeni et. al. [15]. This study showed that different factors such as temperature, freeze-thawing cycles, substrate concentration, the organic solvent used to dissolve the substrate and the time of incubation of enzyme-substrate mixture influenced the final fluorescence intensity. 20 μL of plasma was incubated with 200 μL of buffer (100 mM phosphate buffer, pH 7.5, with 1 mM EDTA, 1 mM DTT and 1 mM sodium azide) for 10 min at 37°C to reach thermal equilibrium. 5 μL of the substrate solution containing 18.4 mM Z-Gly-Pro-4-methylcoumarinyl-7-amide was then added and the mixture incubated at 37°C for 120 min. The reaction was then terminated by the addition of 1000 μL of 1.5 M acetic acid and the release of 7-amino-4-methylcoumarin measured in a fluorimeter (λ_ex_: 370 nm; λ_em_: 440 nm; slit width: 2.5). The substrate solution was prepared by dissolving Z-Gly-Pro-4-methylcoumarinyl-7-amide in 100% 1,4-dioxane and then diluting to 50% (v/v) with incubation buffer. All measurements were carried out in triplicate.

The flourometric assay originally used by Goossens et al [16] was incapable of detecting low PEP activity in CSF. By further developing the procedure of previous work [15] it was possible to achieve a 400% improvement in assay sensitivity and detect PEP in CSF [15]. Triple assays of each sample were carried out. Any variation in results was insignificant, which confirmed the reliability of the procedure. The average for each of the three results was used for the calculations.

### Statistical analysis

The figure for the fluorescence intensity and the children's age (Mean +/- statistical deviation) in the two groups were calculated and plotted on a graph. Mann-Whitney U-test was used to test the difference of mean for PEP activity. Levene's test for equality of variances was used for the comparison between the ASD and non-ASD groups of children. SPSS version 11.2 (Norusis, M.J./SPPS Inc., 2004) was used.

## Results

Basal plasma PEP activities in the control group (n = 15) were between 105.4 and 144.0 fluorescence intensity units (mean 124.4, median 125.6). The activity of PEP in the 18 ASD children ranged from 48.1 to 201.9 fluorescence intensity units (mean 134.1, median 147.6) (Table 1).

**Figure 1:**
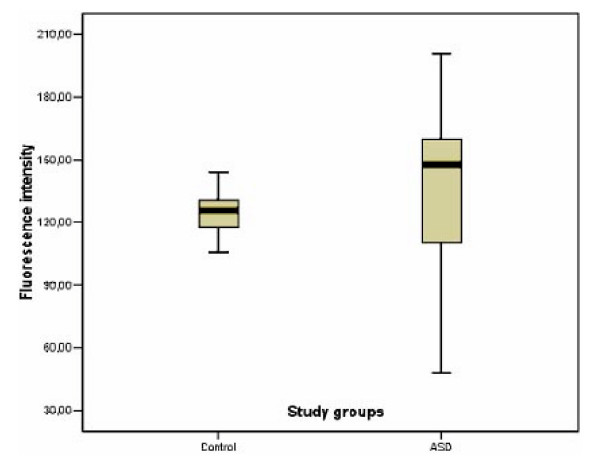
Variation of PEP activity in EDTA plasma in the control group (n = 14), interquartile range (115.8–130.7), and in the ASD group (n = 18), interquartile range (109.3–160.2).

**Figure 2:**
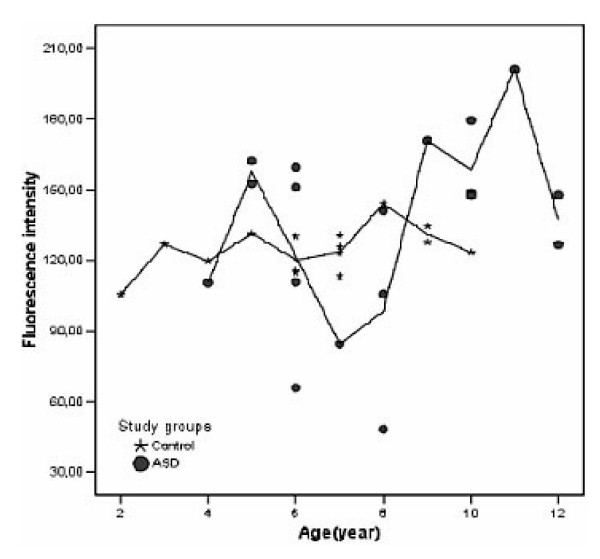
Variation of plasma PEP activity in the children with ASD (n = 18) and in a non-ASD control group (n = 14) associated to age.

The mean level of PEP activity in children with ASD was only slightly higher than that in the controls but the variation of PEP activity was much larger in ASD children than in the controls (SD = 39.9 and 9.6, respectively). The difference was significant (Levene's test for equality of variances yielded F (17,14) = 16.4, p < 0.0005) (Fig. 1). The ASD children were 4–12 years old (mean ± SD: 7.9 ± 2.5), the control group were 2–10 years old (mean ± SD: 6.4 ± 2.2). The variation of enzyme activity is shown in Fig. 2.

## Discussion

Proline endopeptidase, a cytosolic enzyme isolated from human tissues, cleaves different low-molecular-weight neuropeptide hormones such as oxytocin, AVP, TRH, neurotensin, bradykinin and substance P. The neuropeptide hormones, which contain a proline in the carboxyl side of their sequences, act as a substrate for PEP. It has been reported that PEP activity is altered in individuals with depression, mania and schizophrenia [4]. High PEP serum activity has also been reported in patients with PTSD (post-traumatic stress disorder) [17]. The result of this study showed a significantly higher variance of the PEP activity in the group of ASD children. There may be various explanations for this finding, including the heterogeneity of individuals in the ASD group and the effects of pharmaceuticals [4]. The general anaesthesia (GA) may also have an impact on PEP activity. The precise effect of GA on enzyme activity is currently unknown. This question will be addressed in the next study.

This alteration of PEP activity may support our hypothesis that PEP might be involved in the aetiology of ASD. However, our working hypothesis is that ASD can be caused or influenced by external events in early childhood, possibly as result of a genetic predisposition. As a result there may be an inappropriate release of the cytosolic proteolytic enzyme PEP into the circulating blood stream and the cerebrospinal fluid (CSF). PEP cleaves different neuropeptides or their precursor molecules leading to an alteration of the concentration of neuropeptides and this may have a negative effect upon proper brain function.

The main deficits of children with ASD include early difficulties with social contact, such as eye contact and social smile [18,19], attention [20], affects [21,22], reciprocity [18,23], turn-taking, timing and answering parents' signals [21]. Many different problems can arise with respect to co-ordination and motor planning [18,24], body tonus deficits [19], and problems with mobility. Children with ASD have a tendency to ignore other people or may even prefer to be alone [19]. They also have difficulties in signalling for attention and they communicate without meeting the gaze of another person [18]. This could be explained by the effects PEP might have on the neuropeptide hormones when cleaving them and interfering with their proper functions in the processes of early brain development.

In three children with ASD, the PEP activity was lower than the mean activity of the control group. Twelve ASD children had higher PEP activity than average and the remaining three had PEP activity equal to that of the control group. This variation may be related to different psychiatric disorder from which the patients were suffering, such as depression or mania. There was no significant gender difference in enzyme activity in the control group, neither was there any great variation but in the ASD group there was a significant variation, randomly distributed between the sexes.

We did not measure PEP activity in the cerebrospinal fluid (CSF) of the ASD children. It might differ from plasma PEP activity. In a previously published article [15] PEP activities was measured in both CSF and EDTA plasma in patients suffering from another neurological disease in order to investigate whether PEP activity differs in these two substances from the same patient. The result showed a variation in PEP activity in plasma compared to CSF. This variation might be caused by a dysfunction in the blood brain barrier (BBB) that might allow substances such as PEP to pass through the barrier and continue its activity in the circulating blood. BBB dysfunction might also be implicated in the variation of the EDTA plasma PEP activity in the children with ASD.

Due to the pioneering nature of this research it is difficult to relate the finding of this study to other biomedical research on ASD. Research is also limited regarding PEP activity associated with other psychiatric disorders. Altered prolyl endopeptidase activity in plasma has been associated with major depressed patients (low levels) and with manic and schizophrenic patients (high levels) [4]. This preliminary finding may indicate an association between altered PEP activity and neuro-psychiatric disorders such as ASDs.

## Conclusion

Our preliminary finding suggests a role for PEP enzyme in the pathophysiology of autism but further research should be conducted to establish its role in the aetiology of psychiatric and neurological disorders, including autism and related spectrum disorders.

## Competing interests

The author(s) declare that they have no competing interests.

## Authors' contributions

NM planned and performed all experiments presented in this study. HA participated in the design of the study. BN and VH analysed data and participated in the preparation of the manuscript. BS, the corresponding author, is the academic supervisor of NM. BS supervised all aspect of the statistical analysis and the writing of the manuscript. BSs particular interest is the relationship between the biomedical aspects and the autistic spectrum disorders.

## Pre-publication history

The pre-publication history for this paper can be accessed here:



## References

[B1] Walter R, Simmons WH, Yoshimoto T (1980). Proline specific endo-and exopeptidases. Mol Cell Biochem.

[B2] Welches WR, Brosnihan KB, Ferrario CM (1993). A comparison of the properties and enzymatic activities of three angiotensin processing enzymes: angiotensin converting enzyme, prolyl endopeptidase and neutral endopeptidase 24.11. Life Science.

[B3] Insel TR (1992). Oxytocin : a neuropeptide for affiliation: evidence from behavioral, receptor autoradiographic and comparative studies. Psychoneuroendocrin.

[B4] Maes M, Goossens F, Scharpé S, Calabrese J, Desnyder R, Meltzer HY (1995). Alteration in plasma prolyl endopeptidase activity in depression, mania, and schizophrenia: effect of antidepressants, mood stabilizers, and antipsychotic drugs. Psychiatry Res.

[B5] American Psychiatric Association (1994). DSM-IV: Diagnostic and statistical Manual of Mental Disorders.

[B6] Bauman MN (1991). Microscopic neuroanatomic abnormalities in autism. Pediat.

[B7] Gillberg C, Steffenburg S, Wahlström J, Gillberg IC, Sjöstedt A, Martinsson T, Liedgren S, Olofsson OE (1991). Autism associated with marker chromosome. J Am Acad Child Adolesc Psychiatry.

[B8] London EA (2000). The environment as an etiologic factor in autism: a new direction for research. Environ Health Perspect.

[B9] Robinson PD, Schutz CK, Macciardi F, White BN, Holden JJ (2001). Genetically determined low maternal serum dopamine beta-hydroxylase levels and the etiology of autism spectrum disorders. Am J Med Genet.

[B10] Singh VK, Singh EA, Warren RP (1997). Hyperserotoninemia and serotonin receptor antibodies in children with autism but not mental retardation. Biol Psychiatry.

[B11] Cook EH, Leventhal BL (1996). The serotonin system in autism. Curr Opin Pediat.

[B12] Scifo R, Cioni M, Nicolosi A, Batticane N, Tirolo N, Quattropani MC, Morale MC, Gallo F, Marchetti B (1996). Opioid-immune interactions in autism: behavioural and immunological assessment during a double-blind treatment with naltrexone. Ann Ist Super Sanita.

[B13] Gillberg C, Terenius L, Lonnerholm G (1985). Endorphin activity in childhood psychosis. Arch Gen Psychiatry.

[B14] Modahl C, Green AL, Fein D, Morris M, Waterhouse L, Feinstein C, Levin H (1998). Plasma Oxytocin Levels in Autistic Children. Biol Psychiatry.

[B15] Momeni N, Yashimoto T, Grubb A (2003). Factors influencing analysis of prolyl endopeptidase in human blood and cerebrospinal fluid: increase in assay sensitivity. Scand J Clin Lab Invest.

[B16] Goossens F, Meester ID, Vanhoof G, Scharpé S (1992). A sensitive method for the assay of serum prolyl endopeptidase. Eur J Clin Chem Clin Biochem.

[B17] Maes M, Lin AH, Bonaccoroso S, Goossens F, Gastel AV, Pioli R, Delmeire L, Scharpé S (1999). Higher serum prolylendopeptidase activity in patients with post-traumatic stress disorder. J Affect Disord.

[B18] Trewarthen C, Aitken K, Papoudi D, Robarths J (1998). Children with autism. Diagnosis and interventions to meet their needs.

[B19] Adrien JL, Lenoir P, Martineau J, Perrot A, Hameury L, Larmande C, Savage D (1993). Blind rating of Early Symptoms of Autism Based upon family Home Movies. J Am Acad Child and Adolesc Psychiatry.

[B20] Maestro S, Muratori F, Cavallo MC, Pei F, Stern D, Golse B, Palacio-Espasa F (2002). Attentional skills during the first 6 months of age in Autism Spectrum Disorders. J Am Acad Child Adolesc Psychiatry.

[B21] Werner E, Dawson G, Osterling J, Dinno N (2000). Recognition of autism spectrum disorders before one year of age: A retrospective study based on home videotapes. J Autism Dev Disord.

[B22] Baranek GT (1999). Autism during infancy: A retrospective video analysis of senso-motor and social behaviors at 9–12 months of age. J Autism Dev Disord.

[B23] Mars AE, Mauk JE, Dowrick PW (1998). Symptoms of pervasive developmental disorders as observed in toddlers in prediagnostic home videos of infants and toddlers. J Pediat.

[B24] Teitelbaum P, Teitelbaum O, Nye J, Fryman J, Maurer RG (1998). Movement analysis in infants may be useful for early diagnosis of autism. Proc Natl Acad Sci U S A.

